# Drug Treatment for Advanced Hepatocellular Carcinoma: First-Line and Beyond

**DOI:** 10.3390/curroncol29080434

**Published:** 2022-08-04

**Authors:** Maple Ye Feng, Landon L. Chan, Stephen Lam Chan

**Affiliations:** 1 Department of Clinical Oncology, Prince of Wales Hospital, The Chinese University of Hong Kong, Hong Kong, China; 2 Department of Oncology, Princess Margaret Hospital, Hong Kong, China; 3 State Key Laboratory of Translational Oncology, Department of Clinical Oncology, The Chinese University of Hong Kong, Hong Kong, China

**Keywords:** advanced hepatocellular carcinoma, systemic therapies, multikinase inhibitors, immunotherapy

## Abstract

Hepatocellular carcinoma (HCC) has high mortality. The option of systemic therapy has increased significantly over the past five years. Sorafenib was the first multikinase inhibitor, introduced in 2007, as a treatment option for HCC, and it was the only effective systemic treatment for more than ten years. It was not until 2017 that several breakthroughs were made in the development of systemic strategies. Lenvatinib, another multikinase inhibitor, stood out successfully after sorafenib, and has been applied to clinical use in the first-line setting. Other multikinase inhibitors such as regorafenib, ramucirumab and cabozantinib, were approved in quick succession as second-line therapies. Concurrently, immune checkpoint inhibitors (ICIs) have readily become established treatments for many solid tumors, including HCC. The most studied ICIs to date, target programmed cell death-1 (PD-1), its ligand PD-L1, and cytotoxic T-lymphocyte-associated protein 4 (CTLA-4). These ICIs have demonstrated efficacy in treating advanced HCC. More recently, combination of bevacizumab and atezolizumab (ICI targeting PD-L1) was approved as the gold-standard first-line therapy. Combination of ICIs with nivolumab and ipilimumab was also approved in the second-line setting for those who failed sorafenib. At the moment, numerous clinical trials in advanced HCC are underway, which will bring continuous change to the management, and increase the survival, for patients with advanced HCC. Our review article: (1) summarizes United States Food and Drug Administration (US FDA) approved systemic therapies in advanced HCC, (2) reports the evidence of currently approved treatments, (3) discusses potential drugs/drug combinations being currently tested in phase III clinical trials, and (4) proposes possible future directions in drug development for advanced HCC.

## 1. Background

HCC has high mortality and a high risk of recurrence after radical treatment. It is recognized as the fourth most common cause of death in cancer patients and has been a healthcare burden globally [1]. Unfavorable outcomes are largely due to the asymptomatic presentation of disease until a later stage, and a lack of effective systemic treatments in advanced HCC [2]. For early-stage disease, curative intent treatments include surgery, radiofrequency ablation, and liver transplant [3,4]. For advanced disease, conventional cytotoxic chemotherapy is ineffective, with limited clinical benefits. In the era before multikinase inhibitors, the overall survival (OS) for HCC patients with advanced disease was expected to be a few months only.

Recently, improved understanding in the molecular biology of hepatocarcinogenesis, and rapid advancement made in diagnostic techniques, have led to the approval of multiple drugs in advanced HCC [5]. Between 2007 and 2016, sorafenib was the only first-line effective therapy [6]. Subsequently, the United States FDA granted approval to four more multikinase inhibitors between 2017 and 2019. Lenvatinib stood out successfully after sorafenib in the REFLECT study, with a noninferiority OS but a higher objective response rate (ORR). Three more multikinase inhibitors, regorafenib, ramucirumab and cabozantinib, were approved in quick succession in the second-line setting [7,8,9,10].

Similar to other cancers, ICIs targeting PD-1, PD-L1 and CTLA-4 have gained momentum in advanced HCC and demonstrated efficacy in a number of clinical trials. These agents work by releasing the immune checkpoints that halt T-cell activation, resulting in the reinvigoration of a functional immune system to attack the cancer cells [11]. The FDA first approved second-line treatment pembrolizumab [12] based on the KEYNOTE-224 in 2018. Based upon the findings of CHECKMATE-040 (cohort 4), nivolumab and ipilimumab combination was approved as a second-line treatment in 2017 [13]. In 2020, first-line bevacizumab combining atezolizumab was approved [14]. All these new therapeutic strategies that emerged in the past few years have substantially revolutionized the treatment for advanced HCC. Our review article summarizes the clinical evidence of FDA-approved systemic therapies for advanced HCC, elucidates potential strategies in phase III clinical trials underway and discusses possible future directions in drug development in advanced HCC.

## 2. Systemic Therapies for Advanced HCC

### 2.1. First-Line Systemic Therapy

#### 2.1.1. Single Agent Multikinase Inhibitor

##### Sorafenib

Until 2017, the only first-line treatment in unresectable HCC was sorafenib. Sorafenib is a multikinase inhibitor which inhibits the activities of kinases and pathways involved in angiogenesis and cell proliferation. It inhibits platelet-derived growth factor receptor (PDGFR), c-KIT, vascular endothelial growth factor receptor (VEGFR) 2/3, RET, RAS/RAF/mitogen-activated protein kinase (MAPK), FLT-3, and Janus kinase (JAK)/signal transducer and activator of transcription protein (STAT) [15,16]. The benefit of first-line sorafenib for inoperable HCC patients with Child A cirrhosis was demonstrated in the multicenter phase III European SHARP trial and the Asia–Pacific trial [6,17]. In the SHARP trial, the sorafenib group showed a better median OS of 10.7 months and time to radiologic progression of 5.5 months, compared with those in the placebo group, which were 7.9 months and 2.8 months, respectively [6]. The time to progression (TTP) and survival benefit were similarly seen in the Asia–Pacific trial which included mostly hepatitis B patients, with median OS and TTP of 6.5 months and 2.8 months in the treatment group, and 4.2 months and 1.4 months in the placebo group, respectively [17]. However, according to the Response Evaluation Criteria in Solid Tumors (RECIST) criteria, the partial response (PR) was low (SHARP: 2%; Asia–Pacific: 3.3%), and complete response (CR) was not seen in both trials [6,17]. With respect to the benefit based on the etiology of liver disease, the SHARP trial identified that the difference between treatment and placebo group in median OS was most significant in the hepatitis C-HCC group at 6.6 months. The differences in OS were smaller in hepatitis B-HCC at 3.6 months and alcoholic HCC at 2.3 months [18].

Regarding the safety profile for sorafenib, adverse events (AEs) were generally manageable. The most common grade 3/4 AEs included hand–foot syndrome (HFS) (SHARP: 8%; Asia–Pacific: 11%) and diarrhea (SHARP: 8%; Asia–Pacific: 6%) [6,17].

Although sorafenib increases survival in advanced HCC patients, drug resistance is commonly encountered [19]. Clinical evidence supporting the presence of hypoxia is essential in HCC development. The persistent antiangiogenic effect exerted by the long-term use of sorafenib can lead to hypoxia-inducible factors (HIFs)—mediated cellular responses which promote and select resistant cells adaptive to hypoxic microenvironment. Therefore, HIF-1α and HIF-2α overexpression are recognized as poor prognostic markers in HCC patients [19]. 

##### Lenvatinib

Lenvatinib is another multikinase inhibitor which inhibits VEGFR, PDGFR, KIT, RET and fibroblast growth factor receptor (FGFR) activities [20]. In 2018, the REFLECT trial revealed non-inferiority median OS of lenvatinib at 13.6 months compared with 12.3 months in the sorafenib group [21]. Regarding secondary endpoints, determined by investigator review according to modified RECIST (mRECIST), lenvatinib was associated with higher ORR of 24.1%, better PFS of 7.4 months and longer median TTP of 8.9 months. For the sorafenib group, the results were 9.2%, 3.7 months and 3.7 months. A masked independent imaging review according to mRECIST confirmed the above results. Lenvatinib significantly improved ORR at 40.6%, PFS at 7.3 months and median TTP at 7.4 months. In the sorafenib group, the results were 12.4%, 3.6 months and 3.7 months, respectively [21]. In terms of treatment-related toxicities, lenvatinib was associated with more common grade 3/4 hypertension (23% vs. 14%), while sorafenib had more HFS (52% vs. 37% any grade, 11% vs. 3% grade 3 or worse) and alopecia of any grade (25% vs. 3%). 

Based on this finding, lenvatinib is now an alternative first-line multikinase inhibitor for advanced HCC. Japan approved its use in March 2018. Subsequently, it received approval in the U.S. for the same indication in August 2018. The American Society of Clinical Oncology (ASCO), European Society for Medical Oncology (ESMO), National Comprehensive Cancer Network (NCCN) and European Association for the Study of the Liver (EASL) also supported its use in the first-line setting, but limited to individuals with Child A cirrhosis [14,22,23].

#### 2.1.2. Combining ICI with an Anti-VEGF Antibody

##### Atezolizumab and Bevacizumab

Atezolizumab is a PD-L1 blocker, and bevacizumab is a VEGF inhibitor. Both are monoclonal antibodies. This combination is now FDA approved and has superseded sorafenib as the gold-standard first-line treatment in unresectable HCC. This approval was based on the findings from the IMBrave 150 trial which compared atezolizumab and bevacizumab with sorafenib in the first-line setting [24]. The combination therapy demonstrated a significantly better median PFS of 6.8 months vs. 4.3 months for sorafenib. The median OS was also improved to 19.2 months with combination therapy vs. 13.4 months for sorafenib. The ORR was almost threefold better with atezolizumab and bevacizumab (30%), compared with sorafenib (11%) [25]. In the atezolizumab and bevacizumab group, 5.5% achieved CR, and 21.8% achieved PR. There was no CR in the sorafenib group.

The overall grade 3/4 AEs were similar in both groups (atezolizumab–bevacizumab: 57%; sorafenib: 55%), but hypertension (atezolizumab–bevacizumab: 15.2%; sorafenib: 12.2%), proteinuria (atezolizumab–bevacizumab: 3.0%; sorafenib: 0.6%), raised aspartate aminotransferase (AST) (atezolizumab–bevacizumab: 7.0%; sorafenib: 5.1%) and elevated alanine aminotransferase (ALT) (atezolizumab–bevacizumab: 3.6%; sorafenib: 1.3%) were more common with combination therapy [24]. There were no unexpected safety signals. 

In summary, for Child A cirrhosis patients with satisfactory performance status, atezolizumab–bevacizumab is now recommended as the first-line therapy. This is consistent with the ASCO 2020 guideline [14], the Society for Immunotherapy of Cancer 2021 guideline [26], and the recommendation from the EASL [27]. 

##### Atezolizumab and Cabozantinib

The COSMIC-312 phase III trial compared cabozantinib monotherapy 60 mg daily, sorafenib monotherapy 400 mg twice daily, and intravenous atezolizumab 1200 mg every three weeks and cabozantinib 40 mg daily in the first-line setting. It randomized 837 patients into the three groups. According to RECIST v1.1, PFS reviewed by a blinded independent review committee (BIRC) was met at a median follow-up of 15.8 months. Atezolizumab–cabozantinib demonstrated a significantly longer median PFS of 6.8 months and reduced disease progression risk by 37% in comparison with sorafenib with a median PFS of 4.2 months (Hazard ratio (HR) 0.63; 99% confidence interval (CI) 0.44–0.91; *p* = 0.0012). The ORRs were 11%, 3.7% and 6.4% in the atezolizumab-cabozantinib, sorafenib and cabozantinib groups, respectively [28]. However, the median OS for the atezolizumab-cabozantinib group and sorafenib group were similar, at 15.4 months and 15.5 months, respectively (HR 0.90; 96% CI 0.69–1.18; *p* = 0.438). The final analysis did not yield a statistically significant OS benefit for cabozantinib plus atezolizumab group compared with sorafenib [29]. A total of 54% and 32% of patients in the atezolizumab–cabozantinib and sorafenib groups experienced grade 3/4 TRAEs. The most common toxicities included HFS (atezolizumab-cabozantinib: 7.9%; sorafenib: 8.2%), hypertension (atezolizumab–cabozantinib: 7.0%; sorafenib: 6.3%), AST elevation (atezolizumab–cabozantinib: 6.5%; sorafenib: 2.4%) and ALT elevation (atezolizumab–cabozantinib: 6.3%; sorafenib: 1.9%), all of which were less than 10% [30]. In summary, COSMIC-312 demonstrated that atezolizumab–cabozantinib was superior to sorafenib regarding PFS rather than OS in the first-line setting with a manageable safety profile. 

#### 2.1.3. PD-1 and CTLA-4 Antibodies Combination

##### Durvalumab and Tremelimumab

A number of studies demonstrated that prolonged exposures to CTLA-4 inhibitors might not be necessary for sustained anti-tumor effects. Zeynep et al. reported that a single dose of CTLA-4 inhibitor tremelimumab could lead to very long duration of objective anti-tumor responses beyond 12 years in advanced melanoma [31]. A phase Ib study showed tremelimumab 1 mg/kg combined with durvalumab (anti-PD-L1) 20 mg/kg every 4 weeks exerted anti-tumor effect with manageable tolerability in advanced non-small cell lung cancer [32]. Recently, a phase I/II trial evaluated durvalumab (20 mg/kg or 1500 mg) and tremelimumab (1 mg/kg or 75 mg) every 4 weeks for four doses followed by durvalumab 20 mg/kg every 4 weeks alone with tolerable toxicity profile and promising initial efficacy in the second-line setting [33]. Expansion phase II trial studied combination 75 mg tremelimumab (T) plus 1500 mg durvalumab (D) (T-75/D-1500) and T-300/D1500, and the high-dose T-300/D1500 combination group showed the best risk and benefit profile with a median OS and ORR of 18.7 months and 22.7%, respectively. The T-300/D-1500 group was further evaluated as first-line therapy in the phase III HIMALAYA trial which was recently published at the ASCO gastroenterology (GI) 2022 meeting [34]. Treatment-naïve patients with inoperable HCC were randomized into four groups: (1) STRIDE (single T regular interval D) regimen: T-300/D-1500 (one dose) and then D-1500 every four weeks; (2) D-1500 every four weeks; (3) sorafenib 400 mg twice daily; (4) T-75 every four weeks (4 doses) and D-1500 every four weeks (T-75/D). Recruitment to T-75/D was discontinued because a planned analysis demonstrated no significant difference between T-75/D and D-1500. The STRIDE group showed a significantly better 3-year OS benefit of 30.7% compared with D-1500 of 24.7% and sorafenib of 20.2%. STRIDE led to a significantly better OS of 16.4 months compared with sorafenib alone at 13.8 months (HR 0.78; 96% CI, 0.65–0.92; *p* = 0.0035). D showed noninferiority to sorafenib alone regarding OS (16.6 vs. 13.8 months; HR, 0.86; 96% CI, 0.73–1.03). The ORR was 20.1% for the T-300/D-1500, 17% for durvalumab alone, and 5.1% for sorafenib alone. However, there was no significant difference in PFS. Durvalumab was non-inferior to sorafenib with favorable safety [34]. The combination group led to grade 3/4 AEs in 25.8% of patients, durvalumab in 12.9% and sorafenib in 36.9% of patients [34]. Therefore, durvalumab and tremelimumab combination is a promising first-line option for patients who are not suitable for atezolizumab and bevacizumab, such as in the scenario with elevated bleeding risk [35]. 

#### 2.1.4. Summary in First-Line Systemic Therapy

**Figure 1:**
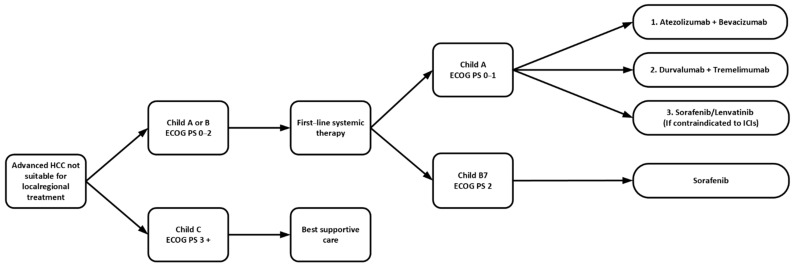
Sequence of first-line systemic therapy in advanced HCC. ECOG PS: Eastern Cooperative Oncology Group Performance Status; ICIs: immune checkpoint inhibitors; HCC: hepatocellular carcinoma.

In short, current guidelines from ASCO and ESMO support the use of first-line combination therapy with atezolizumab–bevacizumab rather than monotherapy with lenvatinib or sorafenib, in unresectable HCC patients with Eastern Cooperative Oncology Group (ECOG) of 0 or 1, Child A cirrhosis without receiving anticoagulant, and following treatment for esophageal varices. For those who are contraindicated to receive bevacizumab, tremelimumab–durvalumab is an alternative option. For patients with Child–Pugh B cirrhosis but no worse than score 7, or when dual immunotherapy is contraindicated, or if clinically the patients were less fit such as marginal performances status 1 or with multiple medical comorbidities with expected poor tolerance to dual immunotherapy, monotherapy with sorafenib or lenvatinib are alternative options. Lenvatinib is only recommended in patients with no worse than Child–Pugh A cirrhosis. In general, lenvatinib has a better toxicity profile compared with sorafenib, such as less HFS and alopecia. It also has a higher ORR, better PFS and longer TTP as per the REFLECT trial. As a result, most clinicians nowadays would prefer to start with lenvatinib if monotherapy is indicated. However, sorafenib might still be preferred given the longer duration of experience and the noninferior median OS demonstrated in the REFLECT trial (Table 1 and Figure 1). 

### 2.2. Second-Line Systemic Therapy

#### 2.2.1. Single-Agent Multikinase Inhibitor

##### Regorafenib

Regorafenib is an oral multikinase inhibitor which shares similarities in structure with sorafenib but demonstrates more profound antiangiogenic effects and tumor growth inhibition (TGI) in preclinical models [36]. Similar to sorafenib, it also targets kinases and cellular pathways involved in angiogenesis and tumor growth, such as the VEGFR, FGFR1, KIT, PDGFR, RET, and BRAF. The US FDA approved second-line use of regorafenib in April 2017 for those who failed sorafenib based upon the RESORCE trial [8]. The trial randomized 573 patients who progressed on sorafenib with ECOG 0–1 and Child A liver function, into either regorafenib or placebo group [8]. Regorafenib showed a significantly better median PFS of 3.1 months, longer median OS of 10.6 months, better ORR of 11% and disease control rate (DCR) of 65%, compared with 1.5 months, 7.8 months, 4% and 36%, respectively, in the placebo group.

In terms of toxicity, regorafenib had more grade 3/4 TRAEs in hypertension (regorafenib 15%, sorafenib 5%), HFS (regorafenib 13%, sorafenib 1%), fatigue (regorafenib 9%, sorafenib 5%), and diarrhea (regorafenib 3%, sorafenib 0%). 68% of patients in the treatment group required dose reduction, while only 31% of patients had dose reduction in the placebo group [8].

##### Cabozantinib

Cabozantinib is another multikinase inhibitor which inhibits VEGFR, MET, RET, AXL and KIT [37]. Exposure to sorafenib may upregulate MET expression and it has been shown as one of the resistance mechanisms to sorafenib in preclinical models [37,38,39]. While low baseline levels of MET were prognostic of better OS, cabozantinib has been shown to associate with better PFS and OS as a second-line post-sorafenib therapy in unresectable HCC regardless of baseline tumor-marker levels. It was granted approval in January 2019 [40]. Its efficacy was demonstrated in the CELESTIAL trial involving 707 patients, which compared cabozantinib with placebo in the second-line post-sorafenib setting [9]. Cabozantinib demonstrated longer median PFS of 5.2 months, improved median OS of 10.2 months and better ORR of 4%. The results were 1.9 months, 8 months and <1% for placebo, respectively. In a later analysis, cabozantinib improved outcomes in those with baseline alpha fetoprotein (AFP) ≥400 ng/mL significantly with median OS of 8.5 months compared with placebo at 5.2 months (HR 0.71; 95% CI 0.54–0.94); while for those with baseline AFP < 400 ng/mL, cabozantinib still showed a trend of a longer median OS to 13.9 months comparing with 10.3 months for placebo, but statistically it did not reach significance (HR, 0.81; 95% CI, 0.62–1.04). As a whole, cabozantinib improved outcomes across the whole spectrum of baseline AFP levels. In addition, it was shown that patients with AFP response in the 8th week were associated with a longer OS at 16.1 months, compared with those without an AFP response at 9.1 months [41].

The most frequent grade 3/4 TRAEs of cabozantinib include HFS (cabozantinib 17%, sorafenib 0%), hypertension (cabozantinib 16%, sorafenib 2%), AST elevation (cabozantinib 12%, sorafenib 7%), fatigue (cabozantinib 10%, sorafenib 4%) and diarrhea (cabozantinib 10%, sorafenib 2%) [40]. 62% and 16% of patients treated with cabozantinib required dose reductions and treatment discontinuation because of TRAEs. The major causes for cabozantinib discontinuation were HFS, fatigue, poor appetite, and diarrhea [9].

#### 2.2.2. Single VEGF Antibody

##### Ramucirumab

Ramucirumab is a recombinant immunoglobulin G subclass 1 (IgG1) class which specifically targets VEGFR-2 and inhibits its activation. Ramucirumab was approved in May 2019 as a second-line option upon progression on sorafenib in those with a high baseline level of AFP ≥ 400 ng/mL [42]. The REACH trial initially failed to show a benefit of ramucirumab [10]. Interestingly, an unplanned subset analysis demonstrated significant better OS of 7.8 months in those with high levels of AFP in the ramucirumab group compared with 4.2 months for placebo [10]. Thereafter, a phase III REACH-2 trial was planned to evaluate the efficacy of ramucirumab in patients who had a baseline AFP ≥ 400 ng/mL after progression on sorafenib [43]. This was a positive study. Compared with placebo, ramucirumab demonstrated significantly better median OS of 8.5 months vs. 7.3 months in the placebo group. Ramucirumab also showed a higher ORR (5% vs. 1%) and overall DCR (60% vs. 39%) [43]. 

Regarding the toxicity profile, grade 3/4 TRAEs were hypertension (ramucirumab 13%; placebo 5%), hyponatremia (ramucirumab 6%; placebo 0%) and elevated AST (ramucirumab 3%; placebo 5%). It is worth mentioning that, unlike other molecularly targeted treatments, ramucirumab does not cause HFS [43].

#### 2.2.3. Single ICI

##### Pembrolizumab

Pembrolizumab (anti-PD-1) is another ICI that had been evaluated in treating advanced HCC. In November 2018, US FDA approved second-line pembrolizumab. In the phase II KEYNOTE-224, pembrolizumab showed benefits in median OS and ORR with 12.9 months and 17%, respectively [12]. The corresponding phase III trial KEYNOTE-240 compared second-line pembrolizumab with best supportive care in unresectable HCC patients who failed first-line sorafenib, however, was a negative trial [44]. Pembrolizumab was associated with numerically better median OS and PFS of 13.9 months and 3 months, respectively, compared with 10.6 months and 2.8 months for best supportive care (BSC). Unfortunately, the pre-specified statistical significance boundary was not reached, so it was technically a negative trial. Yet, pembrolizumab yielded a higher ORR of 18.3% compared with 4.4% for the placebo group, a better median DOR (13.8 months) and more complete responders (six vs. none in the BSC group). 

Recently, the OS data of the phase III trial KEYNOTE-394 was firstly presented at ASCO GI 2022. It tested pembrolizumab in the post-sorafenib setting in an Asian population and reached the conclusion of improved OS, PFS, ORR, DCR, DOR and TTP. Pembrolizumab led to better OS of 14.6 months and PFS of 2.6 months, compared with 13 months and 2.3 months in the placebo group, respectively. Higher ORR (13.7%) and DCR (52.7%) were observed in pembrolizumab group while in placebo group the result was 1.3% and 47.7%, respectively. Median DOR was 23.9 months for pembrolizumab and 5.6 months for BSC, and median TTP was 2.7 months vs. 1.7 months (HR 0.72, 95% CI 0.58–0.90).

14.4–18.6% of patients treated with pembrolizumab and 5.9–7.5% of patients given placebo experienced grade 3/4 TRAEs [44]. The most frequent ones included elevation of AST levels (7%), elevation of alanine aminotransferase (ALT) levels (4%), fatigue (4%) and hyperbilirubinaemia (1%). Immune-related hepatitis was seen in only 3% of patients. There were no hepatitis B or C viral flares identified [12,44]. 

#### 2.2.4. PD-1 and CTLA-4 Antibodies Combination

##### Nivolumab and Ipilimumab

Ipilimumab is an ICI which targets the CTLA-4 molecule, an essential signaling checkpoint required to activate T-cells. When combined with nivolumab, they effectively target two different immune checkpoints and thus release the adaptive immune response. In March 2020, US FDA approved second-line therapy nivolumab plus ipilimumab [45]. The benefits of combined therapy were demonstrated in phase I/II CHECKMATE-040 (cohort 4) that involved 148 sorafenib-treated patients with no worse than a Child A cirrhosis. The trial tested three different regimens: nivolumab 1 mg/kg plus same day ipilimumab 3 mg/kg every three weeks for four cycles then biweekly nivolumab 240 mg (arm A); nivolumab 3 mg/kg plus same day ipilimumab 1 mg/kg every three weeks for four cycles, followed by biweekly nivolumab 240 mg (arm B); or nivolumab 3 mg/kg every two weeks plus ipilimumab 1 mg/kg every six weeks (arm C). The recommended regimen is arm A. The results indicated that the recommended regimen showed the best ORR of 32% [13]. A total of 8% of patients achieved CR and 24% PR. The median DOR was 17 months. The DCR was similar in the three groups. However, trials with a larger sample size are necessary to confirm this result in the future. The recommended regimen is currently being investigated as a first-line therapy in the phase III CHECKMATE-9DW trial. 

The different arms evaluated in this CHECKMATE-040 cohort demonstrated similar patterns of AEs in patients with or without hepatitis B or C, but arm A was associated with more TRAEs. Treatment discontinuation owing to TRAEs occurred in 18% of patients in arm A, 6% in arm B and 2% in arm C. Common immune-mediated side effects included rash in 35%, adrenal insufficiency in 18%, hypothyroidism or thyroiditis in 22%, colitis in 10%, pneumonitis in 10%, and infusion-related reactions in 8% [13]. 

#### 2.2.5. Summary in Second-Line Systemic Therapy 

**Figure 2:**
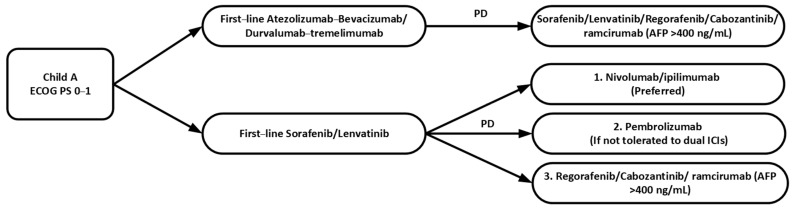
Sequence of second-line systemic therapy in advanced HCC. ECOG PS: Eastern Cooperative Oncology Group Performance Status; ICIs: immune checkpoint inhibitors; PD: disease progression.

The best regimen and optimal sequence of second-line therapy is not well established yet and it depends on patients’ performance status, liver function and the choice of first-line therapy. ASCO guidelines recommend that second-line options for those patients who are initially treated with atezolizumab-bevacizumab or durvalumab-tremelimumab include TKIs such as sorafenib, lenvatinib, regorafenib or cabozantinib [14]. For those patients who progressed on TKIs such as sorafenib or lenvatinib, dual immunotherapy nivolumab-ipilimumab is preferred given the potential for a higher ORR, or pembrolizumab monotherapy as an alternative if the patients are unable to tolerate dual ICIs. If sorafenib or lenvatinib has been chosen as the first-line therapy, regorafenib or cabozantinib can be considered as second-line options if the patients have contraindications to immune checkpoint inhibitors. Ramucirumab is recommended in patients with AFP level more than 400 ng/mL (Table 2 and Figure 2).

## 3. Ongoing Trials in Combination Systemic Treatment

Combining checkpoint inhibitors with multikinase inhibitors, especially anti-angiogenesis therapy, has become mainstream since accelerated FDA approval of atezolizumab and bevacizumab. Multiple studies have demonstrated that the combination of ICIs and TKIs is synergistic by facilitating vascular remodeling and tumor immune stimulation [47,48,49,50]. Lenvatinib and pembrolizumab were studied in a phase Ib trial [51]. This combination showed strong anti-tumor activity with median PFS, OS and ORR of 9.7, 20.4 months and 46%, respectively. Most AEs were manageable by dose modifications. Currently, a direct comparison between this regimen and lenvatinib is underway in the phase III LEAP-002 trial [52]. 

Combination of apatinib (an orally active VEGFR-2 inhibitor) and camrelizumab (SHR1210) (an anti-PD-1 antibody) has also been evaluated in a dose-expansion and escalation phase I trial [53]. The recommended dose with apatinib 250 mg daily and camrelizumab 200 mg every two weeks demonstrated clinical benefits with an ORR of 50%. As a result, this regimen was examined in the phase II RESCUE trial [54]. Treatment-naïve inoperable HCC patients or those who failed or were intolerant to previous TKIs were treated with intravenous camrelizumab 200 mg (body weight ≥ 50 kg) or 3 mg/kg (body weight < 50 kg) every two weeks and apatinib 250 mg daily orally. A total of 70 patients and 120 patients who were predominantly infected with HBV (88.3%) were enrolled in the first-line and second-line setting, respectively. An updated result of the RESCUE trial was recently published in the ASCO meeting 2021. The median time to data cutoff was 29.1 months. In the first-line setting, the median OS was 20.1 months and 2-year OS was 43.3%. In the second-line setting, the median OS was 21.8 months and the 2-year OS was 44.6% [55]. An ongoing phase III trial (NCT03764293) has been designed to directly compare its efficacy with sorafenib in the first-line setting (Table 3). 

## 4. Discussion and Future Perspectives

Since FDA approval for sorafenib in 2007, systemic treatment for HCC has gained considerable momentum with dramatic breakthrough. While multiple multikinase inhibitors have demonstrated efficacy in the treatment of advanced HCC, the introduction of ICIs has truly revolutionized the management of advanced HCC, bringing unprecedented OS benefit and ORR. This is evidenced by the groundbreaking results from the IMbrave150 trial and HIMALAYA trial for the atezolizumab–bevacizumab and durvalumab–tremelimumab combination in the first-line setting, as well as the results from the CHECKMATE-040 trial for the nivolumab–ipilimumab combination in the second-line setting. However, combination therapies are associated with increased toxicities, and each of the above-mentioned regimes have a slightly different toxicity profile. In clinical practice, patients who are very fit such as with performance status 0 to 1 without significant medical comorbidities will be considered for combination therapies, and those who are less fit or with substantial medical comorbidities would be considered for single agent therapy. Furthermore, prior to the start of atezolizumab–bevacizumab therapy, it is important to perform esophago-gastro-duodenoscopy (OGD) to treat any unbanded varices as the dose used for bevacizumab is quite high at 15 mg/kg, which is accompanied by a higher bleeding risk. Therefore, uncertainty remains regarding the best sequence of treatments, in terms of the selection of patient groups who may benefit/tolerate more from one combination over another. Furthermore, there is a paucity of clinical trials performed exploring the role of subsequent treatment in patients who progress on ICIs. This question is currently being explored in several ongoing trials, such as the phase III trial IMbrave 251, exploring atezolizumab–lenvatinib/sorafenib combination in the post-atezolizumab–bevacizumab setting (NCT04770896), and a phase II trial exploring cabozantinib in the post-ICI setting (NCT04588051).

Recently, there have been controversies regarding the use of anti-PD-1 therapy in non-alcoholic steatohepatitis-related HCC (NASH-HCC) patients [56]. Pfisker et al. tested anti-PD-1 treatment in NASH mouse models in both preventive and therapeutic settings [57]. In NASH-mice without tumors, they showed that anti-PD-1 treatment induced hepatic fibrosis and accelerated hepatocarcinogenesis. In NASH-mice bearing HCC, anti-PD-1 treatment did not cause tumor regression. Instead, rather unexpectedly, it accelerated tumor growth. To explore if these findings were also seen similarly in human HCC, they performed a meta-analysis involving 1656 patients from three major trials (IMBrave150, KEYNOTE-240 and CHECKMATE-459), and evaluated the survival outcome of HCC treated with immunotherapy according to the underlying etiology of HCC. They found that immunotherapy only improved survival in viral HCC (HR: 0.64; 95% CI: 0.48–0.94) but not in non-viral HCC (HR: 0.92; 95% CI: 0.77–1.11). However, it was noted that the ORR and PFS of non-viral HCC patients who received anti-PD-1 therapy were similar to those of viral HCC patients. These seemingly contradictory findings have led to several alternative hypotheses, including the heterogeneous population of non-viral HCC and the lack of information regarding downstream treatments [58]. In addition, it is important to note that these inferior outcomes observed for non-viral HCC patients receiving immunotherapy were performed retrospectively. Therefore, this cannot lead to change of clinical practice for patients with HCC based on the etiology of HCC. Further clinical trials with prespecified stratification should be designed to disentangle these controversies.

Questions regarding therapeutic drug resistance and predictive biomarkers remain unanswered. Drug resistance is not uncommon and is recognized as the main cause of treatment failure. Possible mechanisms include EGFR activation, the presence of cancer stem cell (CSC), epithelial–mesenchymal transition (EMT) or the participation of tumor-initiating cells (TIC) [59,60]. During tumorigenesis, activation of EMT signals induce the formation of CSC, resulting in the development of self-renewal and differentiation properties [61]. Long-term sorafenib exposure in human liver cell lines induces EMT with changes in cells’ appearance, loss of E-cadherin and increasing expression of vimentin [62]. Acquisition of EMT in human liver cell lines has been shown to be more resistant to cisplatin, doxorubicin and sorafenib [63]. In another study, β-catenin activation was shown to promote immune escape and resistance to PD-1 ICIs in a genetically engineered mouse model of HCC [64]. These biomarkers are potential candidates to inform treatment decisions, but further studies are needed to translate this bench-side knowledge into bedside. There remains a knowledge gap in understanding tumor biology, as well as an unmet need to develop novel therapies to overcome these resistance mechanisms.

Reliable molecular markers to predict prognosis and treatment response to target therapy, as well as immunotherapy, is lacking. In fact, the diagnosis of HCC is mostly radiological, and the choice of treatment is more or less standardized. In the era of personalized medicine, it is worth exploring biomarkers that can inform treatment decisions. To date, the most extensively studied biomarkers, including microsatellite instability, PD-L1 expression, and tumor mutational burden (TMB) for immunotherapy, have marginal value in HCC [65]. Circulating markers such as AFP, IL-6 and tumor necrosis factor-alpha (TNF-α) have been demonstrated to correlate with HCC treatment outcomes [66,67,68], but more research is needed to verify the result. Recently, gene expression assay has also been explored as a potential biomarker used to predict response to immunotherapy. Haber et al. recently constructed an 11-gene signature that was predictive of response to anti-PD-1 in the first-line setting in patients with inoperable HCC [69].

Finally, potential indirect drug interactions with antibiotics or proton pump inhibitors with ICIs or combination therapies require further investigation [70]. The simultaneous administration of immunotherapy and antibiotics is recognized to negatively influence efficacy in anti-cancer treatment. Several studies have emphasized the strong connection between gut microbiota and ICIs. Microbiota plays a crucial role in intestinal homeostasis and the prevention of systemic inflammation. Antibiotics exert negative impact by inducing dysbiosis, thus altering the systemic anti-tumor response of the immune system during, or in, the first few weeks before starting ICIs [71]. One ongoing trial explores the concomitant use of oral vancomycin, tadalafil and nivolumab in patients with refractory advanced HCC (NCT03785210).

## 5. Conclusions

In conclusion, systemic therapy in advanced HCC has made a breakthrough in the past few years. The introduction of immunotherapy has revolutionized the management of advanced HCC. With the rapid development of molecular biotechnology and precision medicine, many studies are ongoing to better understand the carcinogenesis and drug resistance mechanisms of HCC. We believe with the help of the extensive HCC research and clinical trials, treatment outcomes of advanced HCC will continuously improve in the future.

## Figures and Tables

**Table 1:** First-line systemic therapies for advanced HCC.

Agent	Regimen(s)	Targets	Indication	Sample Size	OS (Months)	OS (HR, 95% CI)	PFS (Months)	ORR (%)	TRAEs (%)	Year of Publication	Year of Approval (if Applicable)
Sorafenib vs. Placebo (SHARP) [6]	Sorafenib 400 mg BID	VEGFR, c-KIT, PDGFR, RET and Ras/Raf/MEK/ERK	First-line as monotherapy	602	Sorafenib vs placebo: 10.7 vs. 7.9	HR 0.69; 95% CI 0.55 to 0.87; *p* < 0.001	5.5 vs. 2.8	PRR 2% vs. 1%	Grade 3/4: diarrhoea 8% vs. 2%, HFS 8% vs. <1%	2007	2007
Sorafenib vs. Placebo (Asia–Pacific) [17]	Sorafenib 400 mg BID	VEGFR, c-KIT, PDGFR, RET and Ras/Raf/MEK/ERK	First-line as monotherapy	226	Sorafenib vs placebo: 6.5 vs. 4.2	HR 0.68; 95% CI 0.50 to 0.93; *p* = 0.014	2.8 vs. 1.4	PRR 3.3%	Grade 3/4: diarrhoea 6%, HFS 11%, fatigue 3%	2007	2007
Lenvatinib vs. Sorafenib (REFLECT) [21]	Lenvatinib 12 mg or 8 mg/d ^#^	VEGFR, PDGFR, FGFR, KIT and RET	First-line as monotherapy	954	Lenvatinib vs. sorafenib: 13.6 vs. 12.3	HR 0.92; 95% CI 0.79 to 1.06	7.4 vs. 3.7	24% vs. 9%	Hypertension 23% vs. 14%, HFS (37% vs. 52% any grade, 3% vs. 11% grade 3/4)	2018	2018 (noninferiority trial)
Bevacizumab (B) and Atezolizumab (A) vs. Sorafenib (IMbrave150) [24]	Atezolizumab IV 1200 mg and bevacizumab IV 15 mg/kg Q3W	VEGF and PD-L1	First-line combination therapy	501	B and A vs. sorafenib: 19.2 vs. 13.4	HR 0.66; 95% CI 0.52 to 0.85; *p* = 0.0009	6.8 vs. 4.3	30% vs. 11%	Grade 3/4: 57% vs. 55% (no statistical difference)	2020	2020
Durvalumab and Tremelimumab (HIMALAYA) [34]	STRIDE: Tremelimumab 300 mg, Durvalumab 1500 mg and Durvalumab 1500 mg Q4W	PDL-1 and CTLA-4	First-line combination therapy	1171	STRIDE: 16.4 D: 16.6 S: 13.8	STRIDE vs. S: HR 0.78; 96% CI 0.65 to 0.92; *p* = 0.0035	STRIDE: 3.8 D: 3.7 S: 4.1 (did not reach statistical significance)	STRIDE: 20.1% D: 17% S: 5.1%	Grade 3/4: STRIDE: 25.8% D: 12.9% S: 36.9%	2022	Under review
Atezolizumab (A) and Cabozantinib (C) (COSMIC-312) [29]	Cabozantinib 60 mg daily, sorafenib 400 BID, atezolizumab IV 1200 mg Q3W and cabozantinib 40 mg daily	PD-L1 and VEGF	First-line combination therapy	837	A and C: 15.4 Sorafenib: 15.5	HR 0.90; 96% CI 0.69 to 1.18; *p* = 0.438	A and C: 6.8 Sorafenib: 4.2	A and C: 11% Sorafenib: 3.7% Cabozantinib: 6.4%	Grade 3/4: A and C: 54%; HFS 7.9%, hypertension 7%, AST elevation 6.5%, ALT elevation 6.3% Sorafenib: 32%; HFS 8.2%, hypertension 6.3%, AST elevation 2.4%, ALT elevation 1.9%	2022	Insignificant OS benefit, not for submission for approval

Abbreviations: BID, twice daily; CI, confidence interval; CTLA-4, cytotoxic T lymphocyte-associated antigen-4; FGFR, fibroblast growth factor receptor; HR, hazard ratio; IV, intravenous; ORR, objective response rate; OS, overall survival; PD-1, programmed cell death-1; PDGFRs, platelet-derived growth factor receptors; PFS, progress free survival; PRR, partial response rates; TRAEs, treatment-related AEs; VEGFRs, vascular endothelial growth factor receptors. ^#^ Lenvatinib oral 12 mg once daily (patients ≥ 60 kg [actual body weight]) or 8 mg once daily (patients < 60 kg [actual body weight]). STRIDE: Tremelimumab 300 mg plus durvalumab 1500 mg (1 dose) plus durvalumab 1500 mg every 4 weeks. D: durvalumab 1500 mg every 4 weeks. S: Sorafenib 400 mg twice daily.

**Table 2:** Second-line systemic therapies for advanced HCC.

Agent	Regimen(s)	Targets	Indication	Sample Size	OS (Months)	OS (HR, 95% CI)	PFS (Months)	ORR (%)	TRAEs (%)	Year of Publication	Year of Approval
Regorafenib vs. Placebo (RESORCE) [8]	Regorafenib 160 mg daily and BSC 1–3W Q4W	VEGFR, FGFR, PDGFR, B-RAF, RET and KIT	Second-line monotherapy in sorafenib-experienced patients	573	Regorafenib vs placebo: 10.6, vs. 7.8	HR 0.63; 95% CI 0.50 to 0.79; *p* < 0·0001	3.1 vs. 1.5	11% vs. 4%	Grade 3/4 HFS 13% vs. 1%, hypertension 15% vs. 5%, diarrhea 3% vs. 0, fatigue 9% vs. 5%	2017	2017
Pembrolizumab (KEYNOTE 224) [12]	IV 200 mg Q 3W or 400 mg Q6W	PD-1	Second-line monotherapy in sorafenib-experienced patients	104	12.9	-	4.9	17%	Grade 3/4 18.6% vs. 7.5%	2018	2018
Pembrolizumab (KEYNOTE 240) [44]	Pembrolizumab IV 200 mg Q 3W or placebo and BSC	PD-1	Second-line monotherapy in sorafenib-experienced patients	413	Pembrolizumab vs. placebo 13.9 vs. 10.6 (no statistical significance)	HR 0.78; 95% CI 0.611 to 0.998; *p* = 0.0238	3 vs. 2.8 (no statistical significance)	18.3% vs. 4.4%	Grade 3/4: AST elevation (7%), ALT elevation (4%), fatigue (4%), hyperbilirubinaemia (1%)	2018	2018
Cabozantinib vs. Placebo (CELESTIAL) [9]	Cabozantinib 60 mg daily	VEGFR, AXL, c-MET, KIT and RET	Second-line monotherapy in sorafenib-experienced patients	707	Cabozantinib vs. placebo: 10.2 vs. 8.0	HR 0.76; 95% CI 0.63 to 0.92; *p* = 0.005	5.2 vs. 1.9	4% vs. <1%	Grade 3/4: hypertension 16% vs. 2%, HFS 17% vs. 0, AST elevation 12% vs. 7%, fatigue 10% vs. 4%, diarrhea 10% vs. 2%	2019	2019
Ramucirumab vs. Placebo (REACH-2) [43]	Ramucirumab IV 8 mg/kg Q2W and BSC	VEGFR2	Second-line monotherapy in sorafenib-experienced patients with AFP ≥ 400 ng/mL	292	Ramucirumab vs. placebo: 8.5 vs. 7.3	HR 0.71; 95% CI 0·531 to 0·949; *p* = 0·0199	2.8 vs. 1.6	5% vs. 1%	Grade 3/4: Hypertension 13% vs. 5%, AST elevation 3% vs. 5%, hyponatremia 6% vs. 0; no HFS	2019	2019
Nivolumab and Ipilimumab (CHECKMATE 040) [13]	Nivolumab IV 1 mg/kg then same day ipilimumab IV 3 mg/kg, Q3W × 4 and nivolumab alone (240 mg Q2W or 480 mg Q4W)	PD-1 and CTLA-4	Second-line combination therapy in sorafenib-experienced patients	148	arm A: 22.8 arm B: 12.5 arm C: 12.7	-	arm A: 17 arm B: 22.2 arm C: 16.6	Best ORR: 32% (arm A)	Adrenal insufficiency 18%, hypothyroidism 22%, rash 35%, pneumonitis 10%, colitis 10%, infusion-related reactions 8%	2020	2020
Pembrolizumab (KEYNOTE 394) [46]	Pembrolizumab IV 200 mg Q 3W or placebo and BSC	PD-1	Second-line monotherapy in sorafenib-experienced patients	453	Pembrolizumab vs. placebo 14.6 vs. 13	HR 0.79; 95% CI 0.63 to 0.99; *p* = 0.018	2.6 vs. 2.3	13.7% vs. 1.3%	Grade 3-5: 14.4% vs. 5.9%	2022	2018

Abbreviations: BSC best supportive care, CI confidence interval, CTLA-4 cytotoxic T lymphocyte-associated antigen-4, FGFR fibroblast growth factor receptor, HR hazard ratio, IV intravenous, ORR objective response rate, OS overall survival, PD-1 programmed cell death-1, PDGFRs platelet-derived growth factor receptors, PFS progress free survival, PRR: partial response rates TRAEs treatment-related AEs, VEGFRs vascular endothelial growth factor receptors. arm A: Give 1 mg/kg of nivolumab and 3 mg/kg of ipilimumab every 3 weeks (4 doses), then 240 mg of nivolumab every 2 weeks. arm B: Give 3 mg/kg of nivolumab and 1 mg/kg of ipilimumab every 3 weeks (4 doses), then 240 mg of nivolumab every 2 weeks. arm C: Give 3 mg/kg of nivolumab every 2 weeks and 1 mg/kg of ipilimumab every 6 weeks, -: not applicable

**Table 3:** Current ongoing key clinical trials in combination systemic treatment of advanced HCC.

Trial Name/ID	Phase	Regimen(s)	Targets	Comparator	Indication	Primary Endpoint(s)	Estimated Primary Completion Date
SHR-1210-III-310	III	Camrelizumab and apatinib	PD-1 and VEGF	Sorafenib	First-line	PFS, OS	21 December
NCT04444167	I/II	AK104 (IV 6 mg/kg Q2W) and Lenvatinib ^#^	PD-1/CTLA-4 and VEGF	N/A	First-line	ORR	22 January
NCT03519997	II	Pembrolizumab (IV 200 mg Q3W) and Bavituximab (IV 3 mg/kg weekly)	PD-1 and anti-phosphatidylserine	N/A	First-line	ORR	22 April
RENOBATE	II	Nivolumab (IV 480 mg Q4W) and Regorafenib (po 80 mg daily for 21 consecutive days Q4W)	PD-1 and VEGF	N/A	First-line	ORR	22 May
NCT04696055	II	Pembrolizumab (IV 400 mg Q6W) and regorafenib (po 90 mg daily 3W on 1W off × 1 then 120 mg daily)	PD-1 and VEGF	N/A	Second-line	ORR	22 May
NCT03418922	I	Nivolumab and lenvatinib	PD-1 and VEGF	N/A	First-line	Safety	22 June
LEAP-002	III	Pembrolizumab (IV 200 mg Q3W) and Lenvatinib ^#^	PD-1 and VEGF	Placebo and Lenvatinib ^#^	First-line	PFS, OS	22 July
NCT03941873	I	Tislelizumab (IV 200 mg Q3W) and Sitravatinib (80 mg/120 mg daily)	PD-1 and VEGF	N/A	First or later lines	Safety	22 August
IMMUNIB	II	Nivolumab (IV 240 MG Q2W up to 36 cycles) and Lenvatinib ^#^	PD-1 and VEGF	N/A	First-line	ORR	22 November
ORIENT-32	II/III	Sintilimab (IV 200 mg Q2W) and IBI305	PD-1 and VEGF	Sorafenib po 400 mg BID	First-line	PFS, OS	22 December
CS1003-305	III	CS1003 and lenvatinib	PD-1 and VEGF	CS1003 placebo and lenvatinib	First-line	PFS, OS	23 June
AMETHISTA	IIIB	Atezolizumab (IV 1200 mg Q3W) and Bevacizumab (IV 15 mg/kg Q3W)	PDL-1 and VEGF	N/A	First-line	Grade 3 or worse NCI CTCAE v.5.0 Bleeding/ Hemorrhage	23 July
NCT04442581	II	Pembrolizumab and Cabozantinib	PD-1 and VEGF	N/A	First-line	ORR	23 September
GOING	I/II	Nivolumab (1.5 mg/kg, 3 mg/kg or 240 mg Q2W) and Regorafenib (160 mg/day 3W on 1W off in the first 8W)	PD-1 and VEGF	N/A	Second-line	Safety	23 December
NCT04183088	II	Tislelizumab and Regorafenib	PD-1 and VEGF	Regorafenib ^	First-line	Safety, ORR, PFS	24 March
CHECKMATE 9DW	III	Nivolumab and ipilimumab	PD-1 and CTLA-4	Sorafenib/lenvatinib	First-line	OS	24 May
ALTN-AK105-III-02	III	AK105 (IV 200 mg Q3W) and anlotinib (po 10 mg daily 2W on 1W off)	PD-1 and VEGF	Sorafenib po 400 mg BID	Second-line	OS	24 June
IMbrave 251	III	Atezolizumab (IV 1200 mg Q3W) and lenvatinib ^#^/sorafenib (po 400 mg BID)	PDL-1 and VEGF	Lenvatinib ^#^/sorafenib (po 400 mg BID)	Second-line	OS	24 October

Abbreviations: BID, twice daily; CTLA-4, cytotoxic T lymphocyte-associated antigen-4; ICI, immune checkpoint inhibitor; IV, intravenous; NCI CTCAE v.5, national cancer institute common terminology criteria for adverse events version 5; ORR, objective response rate; OS, overall survival; PD-1, programmed cell death-1; PFS, progress free survival; VEGF, vascular endothelial growth factor. ^#^ Lenvatinib oral 12 mg once daily (patients ≥ 60 kg (actual body weight)) or 8 mg once daily (patients < 60 kg (actual body weight)). ^ Regorafenib (po 80 mg daily for W1, 120 mg daily for W2, 160 mg daily for W3, dosing-free interval for W4).

## References

[1] Bray F., Ferlay J., Soerjomataram I., Siegel R.L., Torre L.A., Jemal A. (2018). Global cancer statistics 2018: GLOBOCAN estimates of incidence and mortality worldwide for 36 cancers in 185 countries. CA Cancer J. Clin..

[2] Villanueva A., Llovet J.M. (2011). Targeted therapies for hepatocellular carcinoma. Gastroenterology.

[3] Vitale A., Peck-Radosavljevic M., Giannini E.G., Vibert E., Sieghart W., Van Poucke S., Pawlik T.M. (2017). Personalized treatment of patients with very early hepatocellular carcinoma. J. Hepatol..

[4] Kulik L., El-Serag H.B. (2019). Epidemiology and Management of Hepatocellular Carcinoma. Gastroenterology.

[5] Kudo M. (2018). Management of Hepatocellular Carcinoma in Japan as a World-Leading Model. Liver Cancer.

[6] Llovet J.M., Ricci S., Mazzaferro V., Hilgard P., Gane E., Blanc J.F., de Oliveira A.C., Santoro A., Raoul J.L., Forner A. (2008). Sorafenib in advanced hepatocellular carcinoma. N. Engl. J. Med..

[7] Ikeda K., Kudo M., Kawazoe S., Osaki Y., Ikeda M., Okusaka T., Tamai T., Suzuki T., Hisai T., Hayato S. (2017). Phase 2 study of lenvatinib in patients with advanced hepatocellular carcinoma. J. Gastroenterol..

[8] Bruix J., Qin S., Merle P., Granito A., Huang Y.H., Bodoky G., Pracht M., Yokosuka O., Rosmorduc O., Breder V. (2017). Regorafenib for patients with hepatocellular carcinoma who progressed on sorafenib treatment (RESORCE): A randomised, double-blind, placebo-controlled, phase 3 trial. Lancet.

[9] Abou-Alfa G.K., Meyer T., Cheng A.L., El-Khoueiry A.B., Rimassa L., Ryoo B.Y., Cicin I., Merle P., Chen Y., Park J.W. (2018). Cabozantinib in Patients with Advanced and Progressing Hepatocellular Carcinoma. N. Engl. J. Med..

[10] Zhu A.X., Park J.O., Ryoo B.Y., Yen C.J., Poon R., Pastorelli D., Blanc J.F., Chung H.C., Baron A.D., Pfiffer T.E. (2015). Ramucirumab versus placebo as second-line treatment in patients with advanced hepatocellular carcinoma following first-line therapy with sorafenib (REACH): A randomised, double-blind, multicentre, phase 3 trial. Lancet Oncol..

[11] Greten T.F., Lai C.W., Li G., Staveley-O’Carroll K.F. (2019). Targeted and Immune-Based Therapies for Hepatocellular Carcinoma. Gastroenterology.

[12] Zhu A.X., Finn R.S., Edeline J., Cattan S., Ogasawara S., Palmer D., Verslype C., Zagonel V., Fartoux L., Vogel A. (2018). Pembrolizumab in patients with advanced hepatocellular carcinoma previously treated with sorafenib (KEYNOTE-224): A non-randomised, open-label phase 2 trial. Lancet Oncol..

[13] Yau T., Kang Y.-K., Kim T.-Y., El-Khoueiry A.B., Santoro A., Sangro B., Melero I., Kudo M., Hou M.-M., Matilla A. (2020). Efficacy and Safety of Nivolumab Plus Ipilimumab in Patients With Advanced Hepatocellular Carcinoma Previously Treated With Sorafenib: The CheckMate 040 Randomized Clinical Trial. JAMA Oncol..

[14] Gordan J.D., Kennedy E.B., Abou-Alfa G.K., Beg M.S., Brower S.T., Gade T.P., Goff L., Gupta S., Guy J., Harris W.P. (2020). Systemic Therapy for Advanced Hepatocellular Carcinoma: ASCO Guideline. J. Clin. Oncol..

[15] Wilhelm S.M., Carter C., Tang L., Wilkie D., McNabola A., Rong H., Chen C., Zhang X., Vincent P., McHugh M. (2004). BAY 43-9006 Exhibits Broad Spectrum Oral Antitumor Activity and Targets the RAF/MEK/ERK Pathway and Receptor Tyrosine Kinases Involved in Tumor Progression and Angiogenesis. Cancer Res..

[16] Dawkins J., Webster R.M. (2019). The hepatocellular carcinoma market. Nat. Rev. Drug Discov..

[17] Cheng A.L., Kang Y.K., Chen Z., Tsao C.J., Qin S., Kim J.S., Luo R., Feng J., Ye S., Yang T.S. (2009). Efficacy and safety of sorafenib in patients in the Asia-Pacific region with advanced hepatocellular carcinoma: A phase III randomised, double-blind, placebo-controlled trial. Lancet Oncol..

[18] Bruix J., Raoul J.L., Sherman M., Mazzaferro V., Bolondi L., Craxi A., Galle P.R., Santoro A., Beaugrand M., Sangiovanni A. (2012). Efficacy and safety of sorafenib in patients with advanced hepatocellular carcinoma: Subanalyses of a phase III trial. J. Hepatol..

[19] Méndez-Blanco C., Fondevila F., García-Palomo A., González-Gallego J., Mauriz J.L. (2018). Sorafenib resistance in hepatocarcinoma: Role of hypoxia-inducible factors. Exp. Mol. Med..

[20] Al-Salama Z.T., Syed Y.Y., Scott L.J. (2019). Lenvatinib: A Review in Hepatocellular Carcinoma. Drugs.

[21] Kudo M., Finn R.S., Qin S., Han K.H., Ikeda K., Piscaglia F., Baron A., Park J.W., Han G., Jassem J. (2018). Lenvatinib versus sorafenib in first-line treatment of patients with unresectable hepatocellular carcinoma: A randomised phase 3 non-inferiority trial. Lancet.

[22] Vogel A., Cervantes A., Chau I., Daniele B., Llovet J.M., Meyer T., Nault J.C., Neumann U., Ricke J., Sangro B. (2019). Hepatocellular carcinoma: ESMO Clinical Practice Guidelines for diagnosis, treatment and follow-up. Ann. Oncol..

[23] European Association for the Study of the Liver (2018). EASL Clinical Practice Guidelines: Management of hepatocellular carcinoma. J. Hepatol..

[24] Finn R.S., Qin S., Ikeda M., Galle P.R., Ducreux M., Kim T.Y., Kudo M., Breder V., Merle P., Kaseb A.O. (2020). Atezolizumab plus Bevacizumab in Unresectable Hepatocellular Carcinoma. N. Engl. J. Med..

[25] Finn R.S., Qin S., Ikeda M., Galle P.R., Ducreux M., Kim T.-Y., Lim H.Y., Kudo M., Breder V.V., Merle P. (2021). IMbrave150: Updated overall survival (OS) data from a global, randomized, open-label phase III study of atezolizumab (atezo) + bevacizumab (bev) versus sorafenib (sor) in patients (pts) with unresectable hepatocellular carcinoma (HCC). J. Clin. Oncol..

[26] Greten T.F., Abou-Alfa G.K., Cheng A.L., Duffy A.G., El-Khoueiry A.B., Finn R.S., Galle P.R., Goyal L., He A.R., Kaseb A.O. (2021). Society for Immunotherapy of Cancer (SITC) clinical practice guideline on immunotherapy for the treatment of hepatocellular carcinoma. J. Immunother. Cancer.

[27] Bruix J., Chan S.L., Galle P.R., Rimassa L., Sangro B. (2021). Systemic treatment of hepatocellular carcinoma: An EASL position paper. J. Hepatol..

[28] Exelixis Announces Detailed Results from Phase 3 COSMIC-312 Pivotal Trial of Cabozantinib in Combination with an Immune Checkpoint Inhibitor in Patients with Previously Untreated Advanced Liver Cancer at ESMO Asia Virtual Oncology Week 2021. https://www.businesswire.com/news/home/20211120005130/en/Exelixis-Announces-Detailed-Results-from-Phase-3-COSMIC-312-Pivotal-Trial-of-Cabozantinib-in-Combination-with-an-Immune-Checkpoint-Inhibitor-in-Patients-with-Previously-Untreated-Advanced-Liver-Cancer-at-ESMO-Asia-Virtual-Oncology-Week-2021.

[29] Kelley R.K., Yau T., Cheng A.L., Kaseb A., Qin S., Zhu A.X., Chan S., Sukeepaisarnjaroen W., Breder V., Verset G. (2022). VP10-2021: Cabozantinib (C) plus atezolizumab (A) versus sorafenib (S) as first-line systemic treatment for advanced hepatocellular carcinoma (aHCC): Results from the randomized phase III COSMIC-312 trial. Ann. Oncol..

[30] Cammarota A., Zanuso V., D’Alessio A., Pressiani T., Personeni N., Rimassa L. (2022). Cabozantinib plus atezolizumab for the treatment of advanced hepatocellular carcinoma: Shedding light on the preclinical rationale and clinical trials. Expert Opin. Investig. Drugs.

[31] Eroglu Z., Kim D.W., Wang X., Camacho L.H., Chmielowski B., Seja E., Villanueva A., Ruchalski K., Glaspy J.A., Kim K.B. (2015). Long term survival with cytotoxic T lymphocyte-associated antigen 4 blockade using tremelimumab. Eur. J. Cancer.

[32] Antonia S., Goldberg S.B., Balmanoukian A., Chaft J.E., Sanborn R.E., Gupta A., Narwal R., Steele K., Gu Y., Karakunnel J.J. (2016). Safety and antitumour activity of durvalumab plus tremelimumab in non-small cell lung cancer: A multicentre, phase 1b study. Lancet Oncol..

[33] Kelley R.K., Abou-Alfa G.K., Bendell J.C., Kim T.-Y., Borad M.J., Yong W.-P., Morse M., Kang Y.-K., Rebelatto M., Makowsky M. (2017). Phase I/II study of durvalumab and tremelimumab in patients with unresectable hepatocellular carcinoma (HCC): Phase I safety and efficacy analyses. J. Clin. Oncol..

[34] Abou-Alfa G.K., Chan S.L., Kudo M., Lau G., Kelley R.K., Furuse J., Sukeepaisarnjaroen W., Kang Y.-K., Dao T.V., Toni E.N.D. (2022). Phase 3 randomized, open-label, multicenter study of tremelimumab (T) and durvalumab (D) as first-line therapy in patients (pts) with unresectable hepatocellular carcinoma (uHCC): HIMALAYA. J. Clin. Oncol..

[35] Maestri M., Pallozzi M., Santopaolo F., Cerrito L., Pompili M., Gasbarrini A., Ponziani F.R. (2022). Durvalumab: An investigational agent for unresectable hepatocellular carcinoma. Expert Opin. Investig. Drugs.

[36] Wilhelm S.M., Dumas J., Adnane L., Lynch M., Carter C.A., Schütz G., Thierauch K.-H., Zopf D. (2011). Regorafenib (BAY 73-4506): A new oral multikinase inhibitor of angiogenic, stromal and oncogenic receptor tyrosine kinases with potent preclinical antitumor activity. Int. J. Cancer.

[37] Xiang Q., Chen W., Ren M., Wang J., Zhang H., Deng D.Y.B., Zhang L., Shang C., Chen Y. (2014). Cabozantinib Suppresses Tumor Growth and Metastasis in Hepatocellular Carcinoma by a Dual Blockade of VEGFR2 and MET. Clin. Cancer Res..

[38] Rimassa L., Assenat E., Peck-Radosavljevic M., Pracht M., Zagonel V., Mathurin P., Rota Caremoli E., Porta C., Daniele B., Bolondi L. (2018). Tivantinib for second-line treatment of MET-high, advanced hepatocellular carcinoma (METIV-HCC): A final analysis of a phase 3, randomised, placebo-controlled study. Lancet Oncol..

[39] Firtina Karagonlar Z., Koc D., Iscan E., Erdal E., Atabey N. (2016). Elevated hepatocyte growth factor expression as an autocrine c-Met activation mechanism in acquired resistance to sorafenib in hepatocellular carcinoma cells. Cancer Sci..

[40] Rimassa L., Kelley R.K., Meyer T., Ryoo B.Y., Merle P., Park J.W., Blanc J.F., Lim H.Y., Tran A., Borgman-Hagey A.E. (2019). Outcomes based on plasma biomarkers for the phase III CELESTIAL trial of cabozantinib (C) versus placebo (P) in advanced hepatocellular carcinoma (aHCC). Ann. Oncol..

[41] Kelley R.K., Meyer T., Rimassa L., Merle P., Park J.W., Yau T., Chan S.L., Blanc J.F., Tam V.C., Tran A. (2020). Serum Alpha-fetoprotein Levels and Clinical Outcomes in the Phase III CELESTIAL Study of Cabozantinib versus Placebo in Patients with Advanced Hepatocellular Carcinoma. Clin. Cancer Res..

[42] FDA Approves Ramucirumab for Hepatocellular Carcinoma. https://www.fda.gov/drugs/resources-information-approved-drugs/fda-approves-ramucirumab-hepatocellular-carcinoma.

[43] Zhu A.X., Kang Y.K., Yen C.J., Finn R.S., Galle P.R., Llovet J.M., Assenat E., Brandi G., Pracht M., Lim H.Y. (2019). Ramucirumab after sorafenib in patients with advanced hepatocellular carcinoma and increased α-fetoprotein concentrations (REACH-2): A randomised, double-blind, placebo-controlled, phase 3 trial. Lancet Oncol..

[44] Finn R.S., Ryoo B.Y., Merle P., Kudo M., Bouattour M., Lim H.Y., Breder V., Edeline J., Chao Y., Ogasawara S. (2020). Pembrolizumab as Second-Line Therapy in Patients with Advanced Hepatocellular Carcinoma in KEYNOTE-240: A Randomized, Double-Blind, Phase III Trial. J. Clin. Oncol..

[45] FDA Grants Accelerated Approval to Nivolumab and Ipilimumab Combination for Hepatocellular Carcinoma. https://www.fda.gov/drugs/resources-information-approved-drugs/fda-grants-accelerated-approval-nivolumab-and-ipilimumab-combination-hepatocellular-carcinoma.

[46] Qin S., Chen Z., Fang W., Ren Z., Xu R., Ryoo B.-Y., Meng Z., Bai Y., Chen X., Liu X. (2022). Pembrolizumab plus best supportive care versus placebo plus best supportive care as second-line therapy in patients in Asia with advanced hepatocellular carcinoma (HCC): Phase 3 KEYNOTE-394 study. J. Clin. Oncol..

[47] Allen E., Jabouille A., Rivera L.B., Lodewijckx I., Missiaen R., Steri V., Feyen K., Tawney J., Hanahan D., Michael I.P. (2017). Combined antiangiogenic and anti-PD-L1 therapy stimulates tumor immunity through HEV formation. Sci. Transl. Med..

[48] Khan K.A., Kerbel R.S. (2018). Improving immunotherapy outcomes with anti-angiogenic treatments and vice versa. Nat. Rev. Clin. Oncol..

[49] Yi M., Jiao D., Qin S., Chu Q., Wu K., Li A. (2019). Synergistic effect of immune checkpoint blockade and anti-angiogenesis in cancer treatment. Mol. Cancer.

[50] Kudo M. (2020). Scientific Rationale for Combined Immunotherapy with PD-1/PD-L1 Antibodies and VEGF Inhibitors in Advanced Hepatocellular Carcinoma. Cancers.

[51] Finn R.S., Ikeda M., Zhu A.X., Sung M.W., Baron A.D., Kudo M., Okusaka T., Kobayashi M., Kumada H., Kaneko S. (2020). Phase Ib Study of Lenvatinib Plus Pembrolizumab in Patients With Unresectable Hepatocellular Carcinoma. J. Clin. Oncol..

[52] Finn R.S., Zhu A.X. (2021). Evolution of Systemic Therapy for Hepatocellular Carcinoma. Hepatology.

[53] Xu J., Zhang Y., Jia R., Yue C., Chang L., Liu R., Zhang G., Zhao C., Zhang Y., Chen C. (2019). Anti-PD-1 Antibody SHR-1210 Combined with Apatinib for Advanced Hepatocellular Carcinoma, Gastric, or Esophagogastric Junction Cancer: An Open-label, Dose Escalation and Expansion Study. Clin. Cancer Res..

[54] Xu J., Shen J., Gu S., Zhang Y., Wu L., Wu J., Shao G., Zhang Y., Xu L., Yin T. (2021). Camrelizumab in Combination with Apatinib in Patients with Advanced Hepatocellular Carcinoma (RESCUE): A Nonrandomized, Open-label, Phase II Trial. Clin. Cancer Res..

[55] Zhang Y., Xu J., Shen J., Gu S., Wu L., Wu J., Shao G., Zhang Y., Xu L., Yin T. (2021). Update on overall survival (OS) of RESCUE: An open-label, phase 2 trial of camrelizumab (C) in combination with apatinib (A) in patients with advanced hepatocellular carcinoma (HCC). J. Clin. Oncol..

[56] Kelley R.K., Greten T.F. (2021). Hepatocellular Carcinoma—Origins and Outcomes. N. Engl. J. Med..

[57] Pfister D., Núñez N.G., Pinyol R., Govaere O., Pinter M., Szydlowska M., Gupta R., Qiu M., Deczkowska A., Weiner A. (2021). NASH limits anti-tumour surveillance in immunotherapy-treated HCC. Nature.

[58] Chan L.L., Chan S.L. (2022). Novel Perspectives in Immune Checkpoint Inhibitors and the Management of Non-Alcoholic Steatohepatitis-Related Hepatocellular Carcinoma. Cancers.

[59] Sun H., Zhu M.S., Wu W.R., Shi X.D., Xu L.B. (2014). Role of anti-angiogenesis therapy in the management of hepatocellular carcinoma: The jury is still out. World J. Hepatol..

[60] Niu L., Liu L., Yang S., Ren J., Lai P.B.S., Chen G.G. (2017). New insights into sorafenib resistance in hepatocellular carcinoma: Responsible mechanisms and promising strategies. Biochim. Biophys. Acta Rev. Cancer.

[61] Singh A., Settleman J. (2010). EMT, cancer stem cells and drug resistance: An emerging axis of evil in the war on cancer. Oncogene.

[62] van Malenstein H., Dekervel J., Verslype C., Van Cutsem E., Windmolders P., Nevens F., van Pelt J. (2013). Long-term exposure to sorafenib of liver cancer cells induces resistance with epithelial-to-mesenchymal transition, increased invasion and risk of rebound growth. Cancer Lett..

[63] Chen X., Lingala S., Khoobyari S., Nolta J., Zern M.A., Wu J. (2011). Epithelial mesenchymal transition and hedgehog signaling activation are associated with chemoresistance and invasion of hepatoma subpopulations. J. Hepatol..

[64] Ruiz de Galarreta M., Bresnahan E., Molina-Sánchez P., Lindblad K.E., Maier B., Sia D., Puigvehi M., Miguela V., Casanova-Acebes M., Dhainaut M. (2019). β-Catenin Activation Promotes Immune Escape and Resistance to Anti-PD-1 Therapy in Hepatocellular Carcinoma. Cancer Discov..

[65] Singal A.G., Hoshida Y., Pinato D.J., Marrero J., Nault J.C., Paradis V., Tayob N., Sherman M., Lim Y.S., Feng Z. (2021). International Liver Cancer Association (ILCA) White Paper on Biomarker Development for Hepatocellular Carcinoma. Gastroenterology.

[66] Chen T., Dai X., Dai J., Ding C., Zhang Z., Lin Z., Hu J., Lu M., Wang Z., Qi Y. (2020). AFP promotes HCC progression by suppressing the HuR-mediated Fas/FADD apoptotic pathway. Cell Death Dis..

[67] Lai S.-C., Su Y.-T., Chi C.-C., Kuo Y.-C., Lee K.-F., Wu Y.-C., Lan P.-C., Yang M.-H., Chang T.-S., Huang Y.-H. (2019). DNMT3b/OCT4 expression confers sorafenib resistance and poor prognosis of hepatocellular carcinoma through IL-6/STAT3 regulation. J. Exp. Clin. Cancer Res..

[68] Tan W., Luo X., Li W., Zhong J., Cao J., Zhu S., Chen X., Zhou R., Shang C., Chen Y. (2019). TNF-α is a potential therapeutic target to overcome sorafenib resistance in hepatocellular carcinoma. EBioMedicine.

[69] Haber P.K., Torres-Martin M., Dufour J.-F., Verslype C., Marquardt J., Galle P.R., Vogel A., Meyer T., Labgaa I., Roberts L.R. (2021). Molecular markers of response to anti-PD1 therapy in advanced hepatocellular carcinoma. J. Clin. Oncol..

[70] Buti S., Bersanelli M., Perrone F., Tiseo M., Tucci M., Adamo V., Stucci L.S., Russo A., Tanda E.T., Spagnolo F. (2021). Effect of concomitant medications with immune-modulatory properties on the outcomes of patients with advanced cancer treated with immune checkpoint inhibitors: Development and validation of a novel prognostic index. Eur. J. Cancer.

[71] Meriggi F., Zaniboni A. (2021). Antibiotics and steroids, the double enemies of anticancer immunotherapy: A review of the literature. Cancer Immunol. Immunother..

